# Classification of Osteosarcoma Based on Immunogenomic Profiling

**DOI:** 10.3389/fcell.2021.696878

**Published:** 2021-07-16

**Authors:** Xinwen Wang, Liangming Wang, Weifeng Xu, Xinwu Wang, Dianshan Ke, Jinluan Lin, Wanzun Lin, Xiaochun Bai

**Affiliations:** ^1^The Third Affiliated Hospital of Southern Medical University, Guangzhou, China; ^2^Guangdong Provincial Key Laboratory of Bone and Joint Degeneration Diseases, Guangzhou, China; ^3^Department of Orthopedics, The Second Affiliated Hospital of Fujian Medical University, Quanzhou, China; ^4^Department of Medical Oncology, The Affiliated Cancer Hospital of Zhengzhou University, Zhengzhou, China; ^5^Department of Orthopedics, The First Hospital of Putian City, Putian, China; ^6^Department of Orthopedics, Jiangmen People’s Hospital, Jiangmen, China; ^7^Department of Orthopedics, The First Affiliated Hospital of Fujian Medical University, Fuzhou, China; ^8^Department of Radiation Oncology, Shanghai Proton and Heavy Ion Center, Fudan University Cancer Hospital, Shanghai, China

**Keywords:** osteosarcoma, immune subtype, immune checkpoint inhibitors, TYROBP, prognosis

## Abstract

Accumulating evidence has supported that osteosarcoma is heterogeneous, and several subtypes have been identified based on genomic profiling. Immunotherapy is revolutionizing cancer treatment and is a promising therapeutic strategy. In contrast, few studies have identified osteosarcoma classification based on immune biosignatures, which offer the optimal stratification of individuals befitting immunotherapy. Here, we classified osteosarcoma into two clusters: immunity high and immunity low using the single-sample gene-set enrichment analysis and unsupervised hierarchical clustering. Immunity_H subtype was associated with high immune cells infiltration, a favorable prognosis, benefit to immunotherapy, high human leukocyte antigen gene expression, and activated immune signal pathway indicating an immune-hot phenotype. On the contrary, the Immunity_L subtype was correlated with low immune cell infiltration, poor prognosis, and cancer-related pathway, indicating an immune-cold phenotype. We also identified TYROBP as a key immunoregulatory gene associated with CD8^+^ T cell infiltration by multiplex immunohistochemistry. Finally, we established an immune-related prognostic model that predicted the survival time of osteosarcoma. In conclusion, we established a new classification system of osteosarcoma based on immune signatures and identified TYROBP as a key immunoregulatory gene. This stratification had significant clinical outcomes for estimating prognosis, as well as the immunotherapy of osteosarcoma patients.

## Introduction

Osteosarcoma, originating from mesenchymal stem cells, is the most common primary malignancy of bone (ElKordy et al., 2018). With the advancement of surgical resection, radiotherapy, and neoadjuvant chemotherapy, the survival rate of osteosarcoma patients has increased up to 60–70% (Kansara et al., 2014; Simpson and Brown, 2018). However, the prognosis of patients with recurrent, metastatic, or unresectable osteosarcomas is extremely poor. The long-term survival rate for patients with primary osteosarcoma is approximately 65%, whereas it is less than 20% for patients with metastatic osteosarcomas (Durfee et al., 2016; Whelan and Davis, 2018). Hence, a novel therapeutic strategy for advanced sarcomas should be developed to improve the outcome of osteosarcomas. Recently, cancer immunotherapy has received tremendous attention for the treatment of numerous refractory malignancies (Baxevanis et al., 2009; Yang, 2015; Emens et al., 2017; O’Donnell et al., 2019; Lin et al., 2020). Hence, it is worth considering immunotherapy for osteosarcoma, as the treatment options for this disease are still limited.

Accumulating evidence has suggested that osteosarcoma tends to be susceptible to immunotherapy (Chen et al., 2021). Osteosarcoma tumors have a high proportion of CD8^+^ invading lymphocytes relative to other sarcoma subtypes, and the number of infiltrating immune cells associates positively with overall survival (OS) (Wang et al., 2016). Besides, some osteosarcomas have an elevated programmed cell death protein-1 ligand (PD-L1) expression, with a high level of genomic instability, indicating prospective sensitivity to programmed cell death protein-1 (PD-1)/PD-L1 cascade suppressors (Shen et al., 2014). Although some patients benefit from different immunotherapeutic interventions, the majority fail to experience benefit. Thus, considering the low response rate, novel and accurate classification methods should be developed to identify patients befitting immunotherapy.

Here, we performed the single-sample gene-set enrichment analysis (ssGSEA) of the transcriptome data of 29 immune gene sets and established a novel classification of osteosarcoma. We also tried to characterize the subtype-distinct molecular features, including immune signaling cascades, and key genes and to construct and validate the immune-associated prognostic biosignature.

## Materials and Methods

### Datasets

The RNA-seq transcriptome data, as well as the clinicopathological information of osteosarcoma patients, were obtained from TARGET as a training set. Similarly, patients from Gene Expression Omnibus (GSE21257) were abstracted as a validation set.

### Single-Sample Gene-Set Enrichment Analysis

For each osteosarcoma dataset, the levels of enrichment of the 29 immune biosignatures were quantified in each osteosarcoma sample using ssGSEA (Barbie et al., 2009). The ssGSEA enrichment score was obtained by “GSVA” and “GSEABase” packages in R software (version 4.0.0). Gene signatures for each immune cell type and immune pathway were obtained from previously published data, representing the overall immune activity of the tumor microenvironment (He et al., 2018).

### Clustering of Osteosarcoma Samples

Then, we carried out a hierarchical grouping of osteosarcoma based on the 29 immune biosignature enrichment levels (ssGSEA scores). ESTIMATE was carried out to explore the immune score, as well as the tumor purity for each osteosarcoma sample.

### Principal Component Analysis

Principal component analysis (PCA) was performed to compare the transcriptional profiles between the different immune subtypes. The gene names with corresponding expression value and sample information were loaded, and the analysis was conducted by “limma” package using princomp function and visualized by “ggplot2” package in R software (version 4.0.0).

### Identification of Differentially Expressed Immune-Related Genes

The differential expression of immune-related genes was identified using the “limma” R package. Adj. p < 0.05, and | fold change| > 2 were defined as the threshold values. The differentially expressed immune-related genes were visualized by the “ggplot2” package in R software (version 4.0.0).

### Multiplex Immunohistochemistry

Tissue microarray of osteosarcoma specimens (L714901) was obtained from Biotech Company (Xian, China) and used to explore further the association of TYROBP expression with CD8^+^ T cell in the osteosarcoma tumor microenvironment.

Multiplex immunohistochemistry was conducted using the sequential staining cycles as follows. In brief, formalin-fixed, paraffin-embedded osteosarcoma tissue sections were deparaffinized and then underwent microwave treatment in citrate for antigen retrieval. Then, they were blocked with 10% normal goat serum and incubated overnight with primary antibodies: mouse anti-CD8 antibody (1:100, ab17147, Abcam) and rabbit anti-TYROBP antibody (1:200, ab124834, Abcam). Next, sections were incubated with the corresponding horseradish peroxidase-conjugated second antibodies (Abcam, CN) for 30 min at room temperature. The antigenic binding sites were visualized using the tyramide signal amplification dye. Fluorescein isothiocyanate-tyramide (1:1,000, G1235, Servicebio) and Cy3-tyramide (1:1,000, G1235, Servicebio) were applied to each antibody.

### Constitution of a Risk Model

Critical immune-related genes (IRGs) that presented protein–protein interaction (PPI) network were utilized in Lasso Cox regression to calculate the coefficients; the risk-score formula was formulated as:

$$\mathrm{Risk}\,\mathrm{score}=\sum_{i=1}^{\text{N}}\left(E\,x\,p_{i}\times C\,o\,e_{i}\right)$$

where *N* = 2, Expi was the expression value of every five hypoxia genes, whereas the Coei denoted the corresponding multivariable Cox regression coefficient.

### Survival Analyses

Kaplan–Meier curves were used to visualize and compare the OS of osteosarcoma patients in different immunogenomic subtypes *via* survival and survminer R package. A receiver operating characteristic curve was constructed to verify our risk model accuracy *via* the survivalROC R package.

### Visualization of Protein–Protein Interaction Network

We constricted the PPI network using the STRING database Cytoscape software. Cytoscape^1^ constitutes a publicly accessible software web source used in visualizing complex networks, as well as for integrating these with any kind of attribute data. The Cytoscape web portal was used to build a protein cross talk association network and investigated the interaction association of the core IRGs.

## Results

### Immunogenomic Profiling Identifies Two Osteosarcoma Subtypes

**FIGURE 1:**
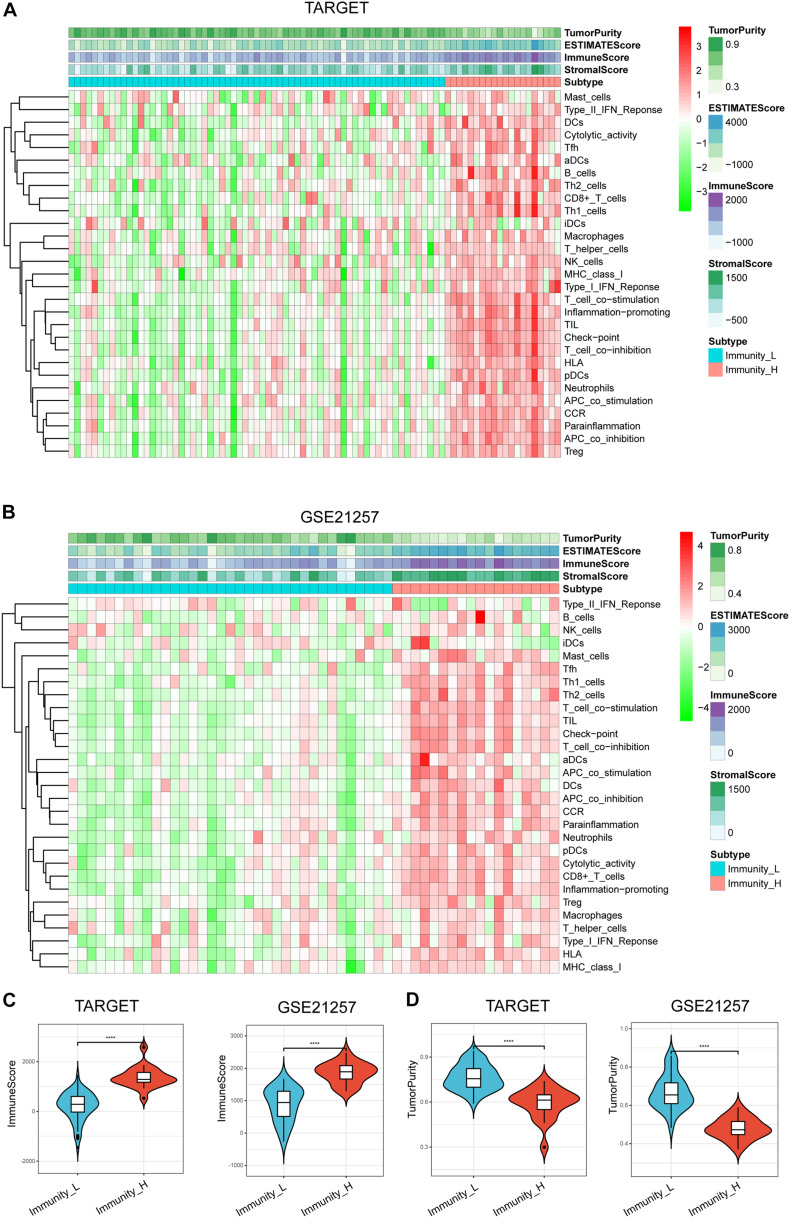
Hierarchical clustering of osteosarcoma identified two immune subtypes in TARGET **(A)** and GSE21257 datasets **(B)**. Correlation between immunogenomic subtypes and immune cell invasion level in immune score **(C)** and tumor purity **(D)**. Violin plots particularly indicated differences in two subtypes. ****p < 0.0001.

We assessed 29 immune-linked gene sets that represented multiple immune cell types, cascades, and functions. The enrichment levels or the activity of immune cells and signaling cascades were quantified in the osteosarcoma samples using ssGSEA analysis. Totally, we hierarchically categorized osteosarcoma in two cohorts (TARGET and GSE21257). Noteworthy, all cohorts presented similar clustering findings, with two clusters clearly being identified. We termed the classes as immunity high (Immunity_H) and immunity low (Immunity_L). Overall, the Immunity_H subtype was correlated with high immune cell infiltration and activated immune pathways indicating an immune-hot phenotype, whereas the Immunity_L subtype was associated with low immune cell infiltration indicating an immune-cold phenotype (Figures 1A,B). Moreover, the immune scores were remarkably elevated in Immunity_H than that in Immunity_L in TARGET and GSE21257 datasets (Figure 1C). Besides, opposite trends were reported with regards to tumor purity between the two subtypes, with tumor purity escalating from Immunity_L to Immunity_H (Figure 1D).

Altogether, these data indicate that Immunity_H has the greatest number of immune cells, which may benefit from immune checkpoint blockade therapy.

### Immunity_H Subtype Is Associated With Immune-Related Signaling Pathways, a Favorable Prognosis and a Potential Benefit to Immunotherapy

**FIGURE 2:**
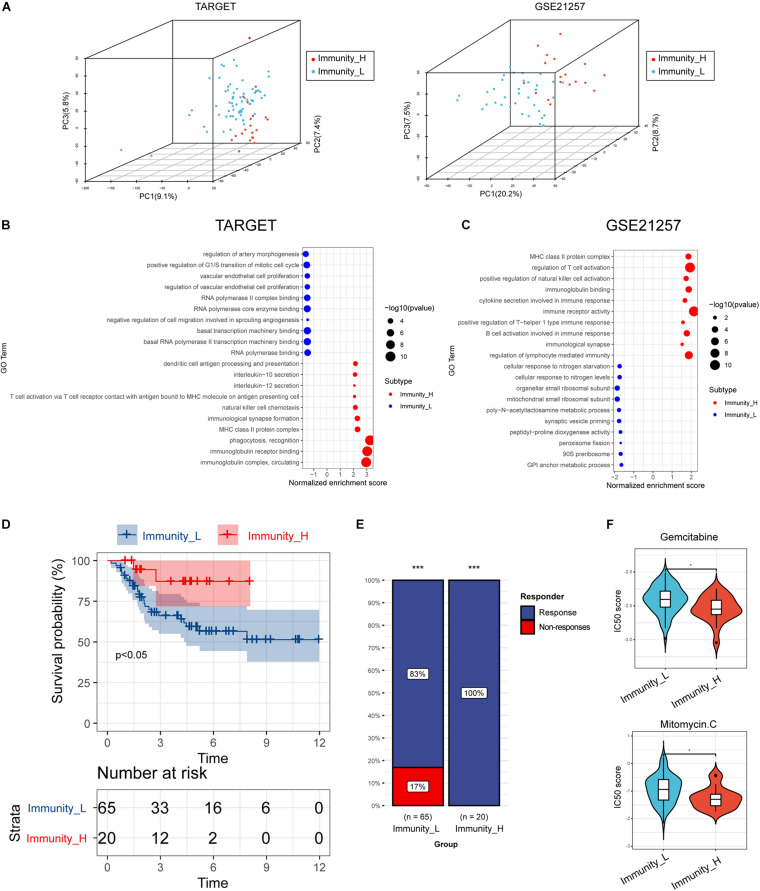
**(A)** Principal component analysis plots. Red dots represent Immunity_H samples, and blue dots represent Immunity_L samples; **(B,C)** GSEA identified differential immune-related signaling cascades enriched in Immunity_H, as well as Immunity_L subtypes in TAGRT and GSE21257 datasets. Size of dots represented -log10 (p-value); **(D)** Kaplan–Meier curves of OS in Immunity_H and Immunity_L subtypes; **(E)** response rate to immunotherapy predicted by TIDE (a computational framework developed to evaluate potential of tumor immune escape from gene expression profiles of cancer samples: http://tide.dfci.harvard.edu/); **(F)** half-maximal inhibitory concentration scores of gemcitabine and mitomycin C in immunity_H and immunity_L subtypes. *p < 0.05, ***p < 0.001, and ****p < 0.0001.

Principal component analysis was then performed to compare the transcriptional profiles between the Immunity_H and Immunity_L subtypes, which displayed a clear distinction. In detail, PCA showed that the samples from the two clusters were well separated from each other (Figure 2A). To further identify relevant signaling cascades activated in the Immunity_H and the Immunity_L groups, we performed a GSEA analysis. Gene sets were differentially enriched in the Immunity_H groups of the TARGET web portal, as they were associated with processes that trigger immunity, such as dendritic cell antigen processing and presentation, interleukin-10 secretion, interleukin-12 secretion, and natural killer cell chemotaxis (Figure 2B). On the contrary, the cancer-linked signaling cascades were hyperactivated in the Immunity_L group, including the positive modulation of G1/S transition of the mitotic cell cycle and vascular endothelial cell proliferation. A similar trend was observed in the GES21257 dataset (Figure 2C). This finding verified the increased immune activity in Immunity_H.

Previous studies showed that osteosarcoma with elevated immune activity and high immune cell infiltration was associated with more favorable clinical outcomes. In line with these pieces of evidence, survival analyses showed that these immune subtypes had distinct clinical outcomes. The Immunity_H subtype had a better survival prognosis than the Immunity_L subtypes (Figure 2D).

We then used TIDE (a computational framework developed to evaluate the potential of tumor immune escape from the gene expression profiles of cancer samples:^2^) to evaluate the potential clinical efficacy of immunotherapy in these immune subtypes. In our results, the Immunity_H subtype had a higher response rate than the Immunity_L subgroup, implying that Immunity_H patients could benefit more from immune checkpoint inhibitor (ICI) therapy (Figure 2E). Besides, chemosensitivity of these subtypes was assessed by the Genomics of Drug Sensitivity in Cancer database, and the results showed that half-maximal inhibitory concentration scores of gemcitabine and mitomycin C in immunity_H were significantly lower than immunity_L subtype, indicating sensitivity to chemotherapy (Figure 2F).

### Differences in Human Leukocyte Antigen Genes and Immune Checkpoint Expression Related to Immune Phenotypes

**FIGURE 3:**
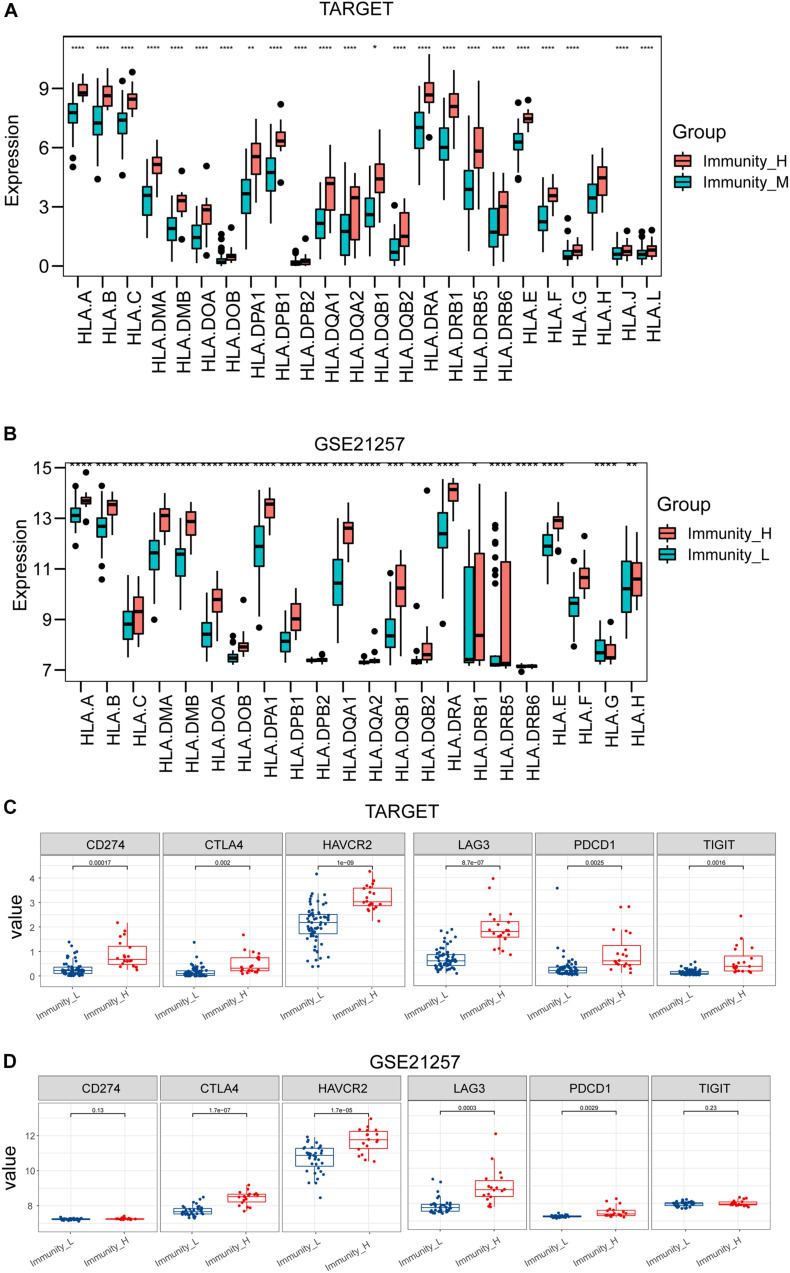
Box plots presented differential expression of HLA genes **(A,B)** and multiple immune checkpoints **(C,D)** between Immune_H and Immune_L subtypes. **p < 0.01, ***p < 0.001, and ****p < 0.0001.

Tumor-related antigen presentation *via* MHC class I complexes is a prerequisite for immune surveillance and is instrumental for the clinical response of immunotherapies targeting immune checkpoints. Hence, we assessed transcript levels of 24 human leukocyte antigen (HLA) genes in TARGET and GSE21257 validation cohorts. Most immune HLA genes exhibited markedly lower expression levels in Immunity_L, indicating impaired antigen presentation on tumor cells as an escape mechanism from immune surveillance (Figures 3A,B). Expression of immune checkpoints has been exploited as predictive biomarkers for checkpoint inhibitor-based immunotherapy; we further compared the differences in immune checkpoints (PD-L1, PD-1, CTLA4, TIM3, LAG3, and TIGIT) related to immunity high and immunity low subtypes. Interestingly, high immune checkpoint expression was observed in the Immunity_H subtype, indicating a potential benefit from checkpoint inhibitor-based immunotherapy (Figures 3C,D).

### Identification of Key Genes Between Immunity_H and Immunity_L Subtypes

**FIGURE 4:**
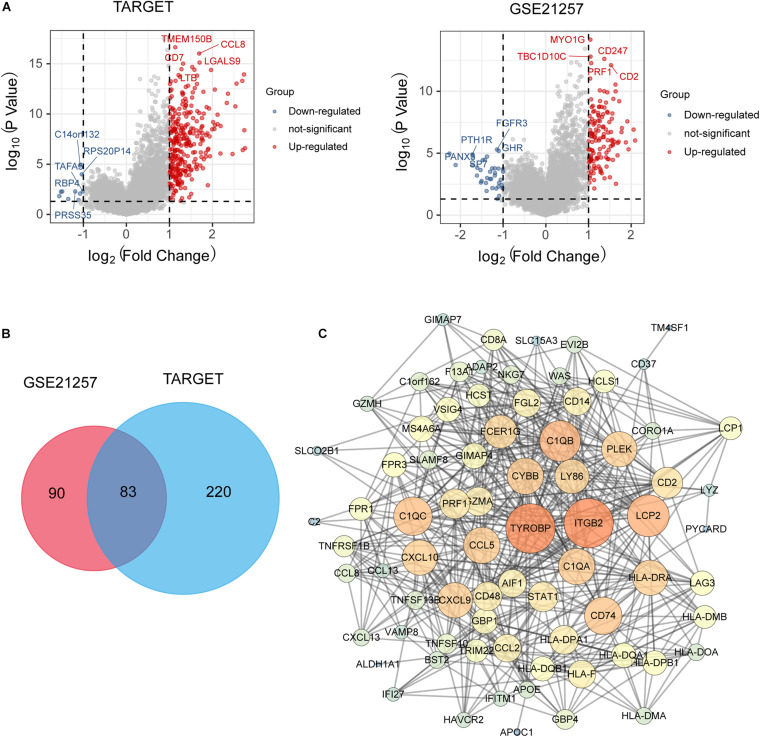
**(A)** Volcano plot presented distribution of differentially expressed genes (DEGs) quantified among Immune_H and Immune_L subtypes with threshold of | log2 Fold change| > 1 and *P* < 0.05 in TARGET and GSE21257 cohort; **(B)** Venn plot showed intersection of differentially expressed genes among Immune_H and Immune_L subtypes; **(C)** PPI network analysis showed interactions among 83 DEGs.

We further identified the differentially expressed genes (DEGs) between Immunity_H and Immunity_L groups and revealed the critical immune-linked genes. A total of 303 DEGs in the TARGET cohort and 173 genes in the GES21257 cohort were identified with an intersection of 83 genes in the two datasets, and most of the genes were upregulated in the Immunity_H group (Figures 4A,B). PPI network was then performed to reveal the key genes associated with the immune subtypes. The top 10 immune-related genes with the highest degrees of interaction were determined, including TYROBP, ITGB2, LCP2, C1QB, C1QC, CD74, HLA-DRA, CXCL10, CCL5, and CXCL9, suggesting their pivotal role in modulating tumor immunity (Figure 4C).

### TYROBP Expression Is Associated With CD8^+^ T Cell Infiltration

**FIGURE 5:**
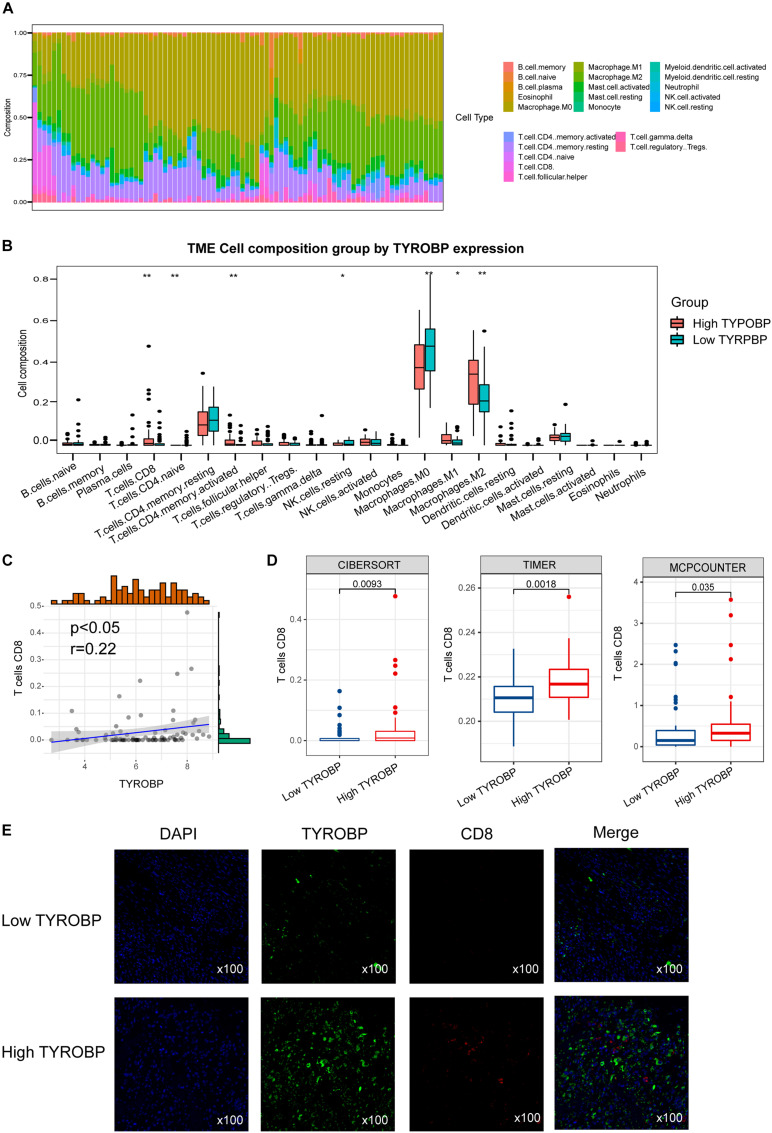
**(A)** Relative proportion of immune infiltration in Immune_H and Immune_L groups; **(B)** box plots visualizing significantly different immune cells between Immune_H and Immune_L groups; **(C)** correlation of TYROBP and CD8^+^ T cell infiltration; **(D)** box plot showed CD8^+^ T cell infiltration level in TYROBP high and low groups; **(E)** multiplex immunohistochemistry validated differentially infiltrated CD8^+^ T cell in TYROBP high and low groups. **p < 0.01, ***p < 0.001, and ****p < 0.0001.

According to PPI analysis, TYROBP showed the highest degrees of interaction with other DEGs, suggesting its important role in driving the immunophenotype. We further investigated the association of TYROBP expression with immune cells in the osteosarcoma tumor microenvironment. Using the CIBERSORT method in combination with the LM22 signature matrix, we calculated differences in the immune infiltration of 22 immune cell types between low- and high-TYROBP groups. Figure 5A summarized the 22 immune cell landscapes among osteosarcoma patients in the TARGET dataset. The results showed that patients with high TYROBP expression had significantly higher proportions of immune cells (e.g., CD8 T cell, CD4 naive T cell, CD4 memory activated T cell, macrophages M0, macrophages M1, and macrophages M2) (Figure 5B). Among the immune cells, CD8^+^ T cells, also known as cytotoxic T cells, play a vital role in antitumor immunity by killing cancer cells. Our results demonstrated that TYROBP expression showed a positive correlation with CD8^+^ cell infiltration (Figure 5C), and a high TYROBP expression group was infiltrated with a high CD8^+^ T cell level (Figure 5D). To further validate the association between TYROBP and CD8^+^ T cells in osteosarcoma, we performed multiplex immunohistochemistry. In line with results from bioinformatics analysis, multiplexed immunohistochemistry showed that high TYROBP expression was associated with high CD8^+^ T cell levels in the tumor microenvironment (Figure 5E).

### Construction and Confirmation of the Immune-Related Prognostic Biosignature

**FIGURE 6:**
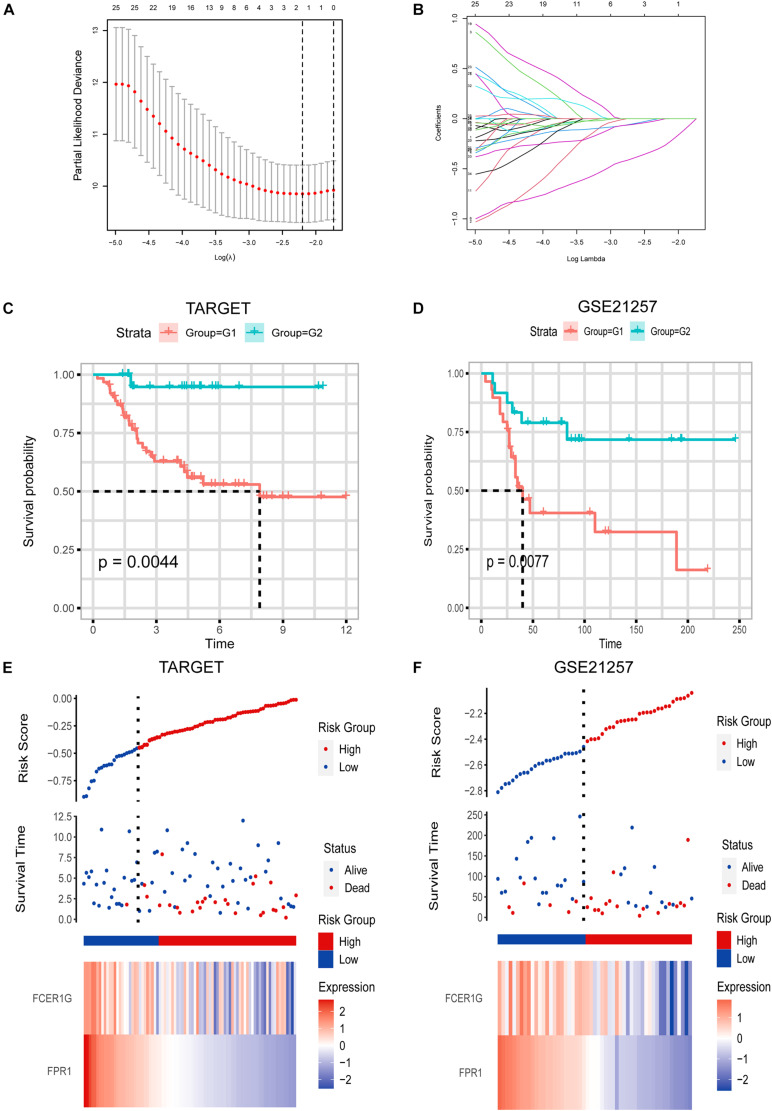
**(A,B)** Lasso Cox analysis uncovered two genes most associated with OS in TARGET dataset. **(C,D)** Kaplan–Meier curves of OS for LGG patients based on IPS in TARGET cohort and GSE21257 cohort. **(E,F)** Risk scores distribution, survival status of each patient, and heatmaps of prognostic four-gene biosignature in TARGET and GSE21257 datasets.

To improve the accuracy and reliability of the predictive model, we treated TARGET as the training set and the GES21257 as validation sets. Lasso Cox regression analysis was subsequently carried out to construct an immune-associated predictive model using 34 critical IRGs presented in the PPI network (Figures 6A,B). A risk-score formula was established as follows: risk score = −0.267 × FPR1–0.001 × FCER1G. As shown in Figures 6A,B, the heatmap showed that the expression level of FPR1 and FCER1G was decreased, accompanied by the higher risk scores. Additionally, we examined the correlation between risk score and survival status (Figure 6C). Our data showed that the number of alive statuses in the low-risk group was markedly higher relative to the high-risk group (Figure 6D).

To explore the prognostic significance of the risk model in osteosarcoma, we performed a Kaplan–Meier analysis. As indicated in Figure 6, the high-risk score was correlated with dismal OS in the TARGET dataset (cutoff values = −0.44), which was further confirmed by the GSE21257 dataset (cutoff values = −2.4) (Figure 6E).

## Discussion

In this study, we focused on uncovering immune-associated osteosarcoma subtypes through immunogenomic profiling. Our study demonstrates that osteosarcoma could be categorized into immunity low and immunity high subtypes. We established that this categorization was reproducible, as well as predictable. The immunity high osteosarcoma subtype was enriched with high immune cell infiltration and immunoactive signaling pathway indicating an immune hot phenotype, whereas immunity low was associated with low immune cell infiltration and immunosuppressive signaling pathway indicating an immune cold phenotype. Moreover, we developed and validated an immune-related prognostic signature.

Immunity_H had a higher level of immune cell invasion, as well as antitumor immune activities, such as high levels of immunoactivated cell infiltration, and activated immune signaling pathway. This analysis validated the increased antitumor immune activity in the Immunity_H subtype. Indeed, accumulating evidence has documented that the density and level of tumor-infiltrating lymphocytes are positively correlated with survival prognosis in multiple cancers (Hendry et al., 2017; Jiang et al., 2017; Wang et al., 2020; Yao et al., 2017).

The HLA genes code for MHC I, as well as MHC II molecules, which deliver pathogen-originated short peptides to T cells and trigger an adaptive immune response (Apanius et al., 2017; Danchin et al., 2004). Previous researches have reported that the expression of HLA may regulate ICI response in metastatic melanoma (Chowell et al., 2018; Garrido et al., 2016; O’Donnell et al., 2019). The study demonstrated that elevated tumor-distinct expression of MHC-I was pivotal for the response to therapy with anti-CTLA-4. In the meantime, tumor-specific MHC-II expression was critical for the response to the therapy with anti-PD-1 (Johnson et al., 2016). Additionally, recent studies have identified predictive biomarkers (e.g., PD-L1, PD-1, and CTLA-4) that can be exploited to predict response to ICI therapy (Gibney et al., 2016; Lin et al., 2021). In our study, in the Immunity_H subtype, the HLA genes and immune checkpoint expression levels were also the highest compared with the Immunity_L subtype. In contrast, we revealed that Immunity_High exhibited strong immune activity given the high immune invasion level, percentage of distinct tumor-infiltrating lymphocytes, HLA richness, and level of expression and exhibited relatively improved clinical outcomes.

We also identified several key genes with the highest interaction degrees between Immune_H and Immunity_L subtypes, including TYROBP, ITGB2, LCP2, C1QB, C1QC, CD74, HLA-DRA, CXCL10, CCL5, and CXCL9. Previous studies have demonstrated that TYROBP, also known as DAP12, is overexpressed in various cancers. Functionally, TYROBP encodes a transmembrane signaling polypeptide on the surface of a variety of immune cells that contains an immunoreceptor tyrosine-based activation motif in its cytoplasmic domain and mediates signaling transductions (Dietrich et al., 2000; Shabo et al., 2013; Töpfer et al., 2015). It has been reported that TYROBP expression is associated with CD8 T cell infiltration in gastric cancer and clear cell renal cell carcinoma (Jiang et al., 2020; Wu et al., 2020). In line with these pieces of evidence, our result showed that TYROBP expression is positively correlated with CD8 T cell infiltration in the osteosarcoma tumor microenvironment. CD74 plays an important function in the processing of MHC class II antigens through the stabilization of peptide-free class II alpha/beta heterodimers in a complex shortly following their synthesis and directs the transportation of the complex to the endosomal/lysosomal system from the endoplasmic reticulum (Badve et al., 2002; Denzin et al., 1996). High CD74 expression in Immune_H indicated a high antigen presentation and is important for the clinical response of immunotherapies. Pro-inflammatory cytokine participates in a wide variety of processes such as differentiation, chemotaxis, activation of peripheral immune cells, and regulation of immune cell growth (Morrell et al., 2014). CXCL10 is an important pro-inflammatory cytokine and has been reported to mediate the mobilization of tumor-inhibitory CXCR3^+^ T cells, as well as natural killer cells, into solid cancers (Tokunaga et al., 2018). Respectively, a high CXCL10 concentration is correlated with a higher immune cell infiltration and better survival in several malignancies (Bronger et al., 2016).

In our research, we established a novel classification of osteosarcoma based on immunogenomic profiling. We also construct and validate the immune-associated prognostic signature. This classification of osteosarcoma based on immunogenomic profiling may provide valuable information for immunotherapy strategies in osteosarcoma patients. Besides, we also identified TYROBP as a key immunoregulatory gene. Nevertheless, it should be noted that our findings require further validation *in vitro* or *in vivo* and a larger sample cohort. Our findings should be interpreted with this limitation in mind.

In conclusion, we established a novel osteosarcoma classification based on the differences in the transcriptome of 29 immune biosignatures in tumor samples. This classification had significant clinical outcomes for estimating the prognosis, as well as the responsiveness of immunotherapy.

## Data Availability Statement

The datasets presented in this study can be found in online repositories. The names of the repository/repositories and accession number(s) can be found in the article/supplementary material.

## Ethics Statement

The studies involving human participants were reviewed and approved by Ethics Review Committee of the Third Affiliated Hospital of Southern Medical University. Written informed consent for participation was not required for this study in accordance with the national legislation and the institutional requirements.

## Author Contributions

XeW, LW, and WX: conceptualization, methodology, and writing. XuW: software. DK: validation. JL: data curation. WL and XB: project administration and funding acquisition. All authors have read and agreed to the published version of the manuscript.

## Conflict of Interest

The authors declare that the research was conducted in the absence of any commercial or financial relationships that could be construed as a potential conflict of interest.

## Footnotes

1 https://cytoscape.org/

2 http://tide.dfci.harvard.edu/
